# Refractory Angioedema in a Patient with Systemic Lupus Erythematosus

**Published:** 2015-07

**Authors:** Zahra Habibagahi, Jamshid Ruzbeh, Vahide Yarmohammadi, Malihe Kamali, Mohammad Hassan Rastegar

**Affiliations:** 1 Department of Rheumatology, Nemazee Hospital, Shiraz University of Medical Sciences, Shiraz, Iran;; 2 Department of Nephrology, Nemazee Hospital, Shiraz University of Medical Sciences, Shiraz, Iran;; 3 Department of Internal Medicine, Nemazee Hospital, Shiraz University of Medical Sciences, Shiraz, Iran

**Keywords:** Systemic lupus erythematosus, Angioedema, Enalapril

## Abstract

Angioedema secondary to C1 inhibitor deficiency has been rarely reported to be associated with systemic lupus erythematosus. A genetic defect of C1 inhibitor produces hereditary angioedema, which is usually presented with cutaneous painless edema, but edema of the genital area, gastrointestinal and laryngeal tracts have also been reported.

In lupus patients, angioedema may be the result of an acquired type of C1 inhibitor deficiency, most probably due to antibody formation directed against the C1 inhibitor molecule. Herein we report a new case of lupus nephritis that developed angioedema and a rapid course of disease progression with acute renal failure and alveolar hemorrhage without response to high dose steroid and plasmapheresis.

## Introduction

Angioedema usually leads to non-pitting edema of the face, lip, mouth, tongue, extremities, and genitalia.^1^ It can also affect airway and intestinal mucosa, causes airway obstruction and intestinal stenosis. Although angioedema is generally a benign and self-limited disease, but it may be life threatening in some situations with mortality rate from 15 to 33%.^2^^,^^3^

Angioedema can occur in patients with decrease serum levels or abnormal function of regulatory complement protein, C1 inhibitor (C1-INH). Both hereditary and acquired forms of C1-INH deficiency have been defined.

Angioedema must be suspected in any patient with typical clinical presentations and history of any new drug exposure or similar previous attacks. Family members with similar history must also be questioned to find hereditary forms of angioedema.

Few cases of acquired angioedema have been reported in patients with systemic lupus erythematosus (SLE), some of them had an amnions course, which had to be intubated and ICU admitted due to airway obstruction.^4^^,^^5^

## Case Report

An 18-year-old girl, a new case of SLE for about 2 months, presented with facial and lower extremity edema in her last outpatient follow up. She had active urine sediment, serum creatinine of 1.4 mg/dl, and 24 hours urine protein of 2500 mg/day. Renal biopsy confirmed lupus nephritis, class IV, based on the International Society of Nephrology/Renal Pathology Society (ISN/RPS), with cellular crescent formation. She was admitted in internal medicine ward for further management.

On the day of admission, she was afebrile, not in respiratory distress, and her blood pressure was 160/95. She had periorbital edema, malar rash, and oral lesion on hard palate with normal tongue and uvula. The rest of the physical examination was unremarkable.

Ward laboratory test results revealed leukocyte count of 1.5×10^ 9 ^/L, absolute lymph count 0.720×10^ 9 ^/L, hemoglobin 8.0 g/dl, and platelet count 79×10^ 9 ^/L. ESR was 78 mm/hr, albumin 2.9 mg/dl with normal values of liver enzymes and alkaline phosphatase. The patient had serum creatinine 1.5 mg/dl. The results of serology tests were as follow: Antinuclear antibody (ANA) 1/360 with homogenous pattern, anti-double strand DNA antibody >240 IU/ml, anticardiolipin antibody 6.2 GPL units/ml (for the first time), and negative anti La, anti Ro, anti beta2glycoproteinI antibodies and lupus anticoagulant. Both C3 and C4 complement levels were low. Chest X-ray was normal.

On the evening of the day she received the second metylprednisolon pulse, developed sever, generalized sub mandibular and neck swelling progressed to stridor and hoarseness. She was afebrile, tachypnic, and had a normal appearing tongue, soft palate, and uvula. Breathing sounds and the other parts of physical examination had no significant change compared to the admission time. Serum level of C1-INH was low. She still had a normal chest X-ray.

Imipenem and vancomycin were started, after this event, enalapril was discontinued, and emergency intubation with fiberoptic bronchoscopy was performed by anesthesiologist due to low oxygen saturation. Severe epiglottis and vocal cord swelling were noted in the anesthesiologist report.

**Figure 1:**
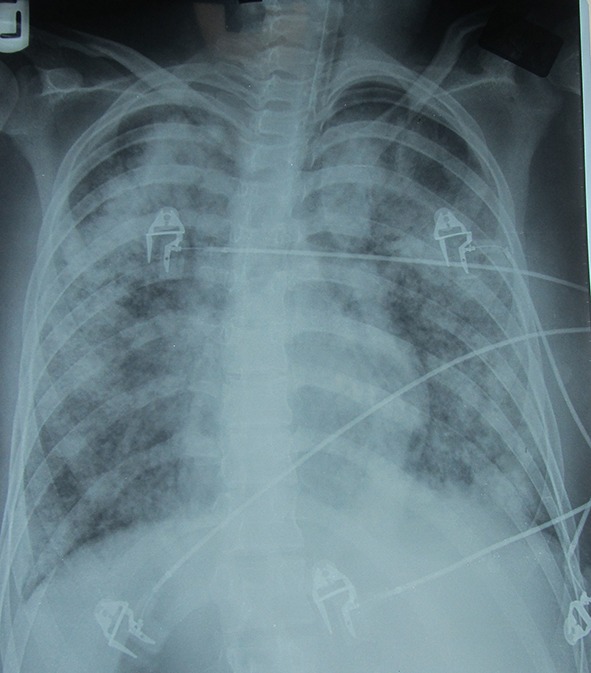
Chest X-ray AP: Bilateral diffuse alveolar infiltration.

In the ICU, antibiotics were continued and she underwent mechanical respiration. Repeated bedside sonography and color Doppler imaging failed to show any collection, hematoma, or vascular thrombosis in the neck area. Few days later, creatinine rose and urine output decreased. Peripheral blood smear was not in favor of thrombotic thrombocytopenia. Dexamethazone (200 mg) and plasmapheresis were started immediately, but she developed bloody secretions from tracheal tube and bilateral coarse rales up to mid part of both lung fields. Chest X-ray showed diffuse bilateral infiltration, suspicious to pulmonary alveolar hemorrhage (figure 1). Finally, she developed cardiac arrest without any response to resuscitation.

## Discussion

Angioedema is defined as localized subcutaneous or submucosal swelling due to extravasation of fluid into interstitial tissues. It affects areas with loose connective tissue. 

Angioedema is classified into two major groups: mast cell mediated, the more common form, and kinin-mediated. Mast cell mediated angioedema is usually associated with urticaria, wheezing, and pruritus after exposure to an identifiable trigger. Kinin-mediated angioedema, however, occurs mostly in the absence of a specific trigger and without clinical signs of mast cell degranulation.^4^

The most well known cause of kinin-mediated angioedema is hereditary C1-INH deficiency. Patients in this group are healthy individuals without underlying disease, have a strong family history, and usually present earlier in life.^6^

Acquired types of C1-INH deficiency resulted from autoantibodies to/or enhanced catabolism of C1-INH have also been described as a cause of angioedema.^7^ This form of angioedema, mostly reported in patients with autoimmune or lymphoproliferative disorders, characterized by adulthood onset and lack of family history.^5^

Angiotensin converting enzyme inhibitors account for a type of kinin-mediated angioedema. These drugs inhibit bradykinin degradation by preventing angiotensin converting enzyme.^8^ ACE inhibitors also unmask C1 inhibitor deficiency.^9^ About 0.1–0.2% of patients may develop angioedema, between several hours to years after drug exposure.^10^ Although most of these cases have mild disease but fatal respiratory problems have also been described.^11^

Our patient was a recently diagnosed case of lupus nephritis according to ACR classification criteria. She developed severe neck swelling and respiratory distress after receiving second metylprednisolon pulse. Our first diagnosis was the infections of the floor of the mouth. Although we did not perform CT scan of the neck, but no response to intravenous antibiotics and negative results of repeated neck sonography were not in favor of an infectious process. Color Doppler imaging also did not show vascular thrombosis, while low serum levels of C3 and C4 complement components, bloody secretions from endotracheal tube and increase in serum creatinine, were all in favor of a rapidly progressive active lupus with acute renal failure. The last chest X-ray in the ICU was compatible with diffuse alveolar hemorrhage, which completed this scenario. We explained low serum level of C1-INH and diffuse epiglottis and vocal cord swelling with acquired angioedema in a setting of an active lupus. Enalapril might also play a role. The patient had no family history or self-history of repeated similar attacks to support a hereditary phenomenon.

Angioedema has been reported in a few cases of SLE. Like our patient, some of them had an intractable course, but most responded to aggressive treatment.^5^^,^^12^ Although most of the previously reported cases of SLE and angioedema had hereditary C1-INH deficiency, the age of presentation in the absence of family history was in favor of an acquired C1-INH deficiency angioedema in this case.^13^^,^^14^

Autoantibodies against C1 inhibitor molecule may induce C1-INH deficiency/dysfunction, leads to hyperactivation of complement classic pathway usually in association with an active underlying autoimmune disease.^15^^,^^16^

Antibodies against C1-INH molecule have not been invariably found in all patients with lupus and angioedema.^17^ Although these patients had low serum concentrations of C1-INH, but unlike our patient, they didn’t have any sign of disease activity.

## Conclusion

Acquired angioedema must be strongly in mind when a patient with lupus, irrespective of disease activity, develops acute neck swelling and shows signs of upper respiratory tract obstruction. Patients show different clinical presentations and variable responses to therapy. This heterogeneity may be due to different mechanisms of angioedema in this autoimmune disease. 

## References

[1] Nzeako UC, Frigas E, Tremaine WJ (2001). Hereditary angioedema: a broad review for clinicians. Arch Intern Med.

[2] Fay A, Abinun M (2002). Current management of hereditary angioedema (C1 esterase inhibitor deficiency). J Clin Pathol.

[3] Markovic SN, Inwards DJ, Frigas EA, Phyliky RP (2000). Acquired C1 esterase inhibitor deficiency. Ann Intern Med.

[4] Lahiri M, Lim AY (2007). Angioedema and systemic lupus erythematosus – A complementary association?. Ann Acad Med Singapore.

[5] Furlanetto V Jr, Giassi Kde S, Neves Fde S, Zimmermann AF, Castro GR, Pereira IA (2010). Intractable acquired autoimmune angioedema in a patient with systemic lupus erythematosus. Rev Bras Reumatol.

[6] Brown T, Cicardi M, Farkas H, Bork K, Kreuz W, Zingala L (2004). Canadian 2003 International Consensus Algorithm For the Diagnosis, Therapy, and Management of Hereditary Angioedema. J Allergy Clin Immunol.

[7] Cicardi M, Zingale LC, Pappalordo E, Folcioni A, Agostoni A (2003). Autoantibodies and lymphoproliferative diseases in acquired C1-inhibitor deficiencies. Medicine (Baltimore).

[8] Morimoto T, Gandhi TK, Fiskio JM, Segar AC, So JW, Cook EF (2004). An evaluation of risk factors foe adverse drug events associated with angiotensin-converting enzyme inhibitors. J Eval Clin Pract.

[9] Dykewics MS (2004). Cough and angioedema from angiotensin-converting enzyme inhibitors: new insights into mechanisms and management. Curr Opin Allergy Clin Immunol.

[10] Hedner T, Samuelsson O, Lunde H, Lindholm L, Andren L, Wiholm BE (1992). Angio-edema in relation to treatment with angiotensin converting enzyme inhibitors. BMJ.

[11] Assadi FK, Wang HE, Lawless S, McKay CP, Hopp L, Fattori D (1999). Angiotensin converting enzyme inhibitor-induced angioedema: a report of two cases. Pediatr Nephrol.

[12] Ko CH, Ng J, Kumar S, Hurst M (2006). Life threatening angioedema in a patient with systemic lupus. Clin Rheumatol.

[13] Koide M, Shirahama S, Tokura Y, Takigawa M, Hayakawa M, Furukawa F (2002). Lupus erythematosus associated with C1 inhibitor deficiency. J Dermatol.

[14] Frigas E, Nzeako UC (2002). Angioedema, pathogenesis, differential diagnosis, and treatment. Clin Rev Allergy Immunol.

[15] Alsenz J, Bork K, Loos M (1987). Autoantibody mediated acquired deficiency of C1 inhibitor. N Engl J Med.

[16] Hunsicker LG, Ruddy S, Carpenter CB, Schur PH, Merrill JP, Müller-Eberhard HJ (1972). Metabolism of third complement component (C3) in nephritis. Involvement of the classic and alternate (properdin) pathways for complement activation. N Engl J Med.

[17] Nettis E, Colanardi MC, Loria MP, Vacca A (2005). Acquired C1 inhibitor deficiency in a patient with systemic lupus erythematosus: a case report and review of the literature. Eur J Clin Invest.

